# Hsa_circ_101882 promotes migration and invasion of gastric cancer cells by regulating EMT

**DOI:** 10.1002/jcla.23002

**Published:** 2019-08-16

**Authors:** Gui‐Hua Yin, Fu‐Cun Gao, Juan Tian, Wen‐Bo Zhang

**Affiliations:** ^1^ Intensive‐care Unit of Linyi Central Hospital Linyi China; ^2^ Galactophore Department of Linyi Central Hospital Linyi China; ^3^ Ultrasonic Department of Linyi Central Hospital Linyi China

**Keywords:** epithelial‐to‐mesenchymal transition, gastric cancer, hsa_circ_101882, invasion, migration

## Abstract

**Background:**

At present, gastric cancer (GC) is a serious threat to human life and health. Non‐coding circular RNAs (circRNAs) have been found abnormal expression in multiple tumors. However, circRNAs remain largely unknown in tumor progression. In the present study, we mainly examined the expression, function, and molecular mechanisms of a new circRNAs (hsa_circ_101882) in GC.

**Materials and methods:**

The expression of hsa_circ_101882 in GC tissue, corresponding adjacent normal tissues, and GC cell lines was examined by RT‐PCR. The function of hsa_circ_101882 in GC was evaluated by MTT assay, cell migration, and invasion assay, colony formation assay, and flow cytometric assay. The effect of hsa_circ_101882 on epithelial‐to‐mesenchymal transition (EMT)‐related gene expression was detected by RT‐PCR and Western blot.

**Results:**

Hsa_circ_101882 expression levels were significantly increased in GC tissue and GC cell lines. Functionally, low expression of hsa_circ_101882 revealed anti‐tumor effects via inhibiting cell growth, migration, and invasion and promoting cell apoptosis. Mechanically, the dysregulated expression of hsa_circ_101882 affects EMT signaling pathway, which was examined by detecting E‐cadherin, N‐cadherin, vimentin, and Snail expression levels.

**Conclusions:**

Therefore, our research reveals that hsa_circ_101882 is considered a metastasis promoter by activating EMT and may serve as a critical oncogene and potential new biomarker in GC.

## 1 INTRODUCTION

At present, gastric cancer (GC) is a serious threat to human life and health. According to the cancer statistics, 2018, gastric cancer is the fifth most common cancer in the world and the third leading cause of cancer death.^1^ However, GC is not sensitive to radiotherapy and chemotherapy and is prone to recurrence and metastasis. Thus, it is to find the molecular mechanisms of GC that helps establish new diagnostic markers and new therapeutic targets for GC.

Circular RNAs (circRNAs) are a newly identified non‐coding RNAs that covalently linked 3′ and 5′ ends to form a closed loop six and possess high stability.^2^ In recent years, studies have found that circRNAs are closely related to tumor development and migration.^3^, ^4^, ^5^ Notably, it is also reported that circRNAs are also related to circRNA_001569 targeting miR‐145 in the proliferation and invasion of colorectal cancer.^6^ Circular RNA CCDC66 promotes colon cancer growth and metastasis.^7^ Although these circRNAs have been discovered the significant role in GC development and metastasis, there is still a unclear in the regulation mechanism of circRNAs in GC. Therefore, it is necessary to search for regulation mechanism of circRNA in GC.

To research the potential roles of circRNAs in migration and invasion of GC, we performed the differential expressed circRNAs by retrieving the microarray data in the GEO dataset (GSE83521), and normalized microarray data were analyzed by using GEO2R after applying log2 transformation. Through microarray data analysis, we found that hsa_circ_101882 was significantly increased in GC. Hsa_circ_101882 is a newly identified circRNA that is located at chr14: 45623899‐45628483. However, to our best knowledge, it has not been reported the biological function and the mechanism of hsa_circ_101882 in tumor. We detected hsa_circ_101882 expression in GC cell lines and tissues. In addition, we further examined the biological function and the mechanism of hsa_circ_101882 in GC cells.

## 2 MATERIALS AND METHODS

### 2.1 Clinical samples

All 58 pairs of GC tissues and corresponding adjacent normal tissues were obtained at the Lin Yi Central Hospital. All samples were collected according to HIPAA guidelines and approved institutional protocols, and none of the patients had received any preoperative therapy. Written informed consent was obtained from all participants, and the study was approved by the Board and Ethics Committee of Lin Yi Central Hospital.

### 2.2 Cell and culture

Human GC cell lines MGC‐803, HGC‐27, SGC‐7901, and MNK‐45 and normal gastric mucosa cell lines GES were supplied from the Type Culture Collection of Chinese Academy of Sciences (Shanghai, China). Human GC cell lines MGC‐803, HGC‐27, SGC‐7901, and MNK‐45 and normal gastric mucosa cell lines GES were cultured in DMEM (Gibco; Thermo Fisher Scientific, Inc.), supplemented with 10% fetal bovine serum (FBS; Gibco; Thermo Fisher Scientific, Inc.), and maintained at 37℃ in an atmosphere of 5% CO2.

### 2.3 Cell proliferation

The cells (5 000) were seeded into 96‐well plates. After 24 hours, 48 hours, and 72 hours, respectively, cell proliferation was detected by methyl thiazolyl tetrazolium (MTT; Sigma‐Aldrich) assay following the manufacturer's instructions (Biosharp). The absorbance at 490 nm was read on a microplate reader.

### 2.4 Cell cycle and apoptosis assay by flow cytometric

For cell cycle assays, cells were harvested into single cell suspensions after trypsinization and fixed with cold ethanol for 1 hour at 4°C. Cells were then treated with RNase A for 30 minutes and labeled with PI for 15 minutes. For apoptosis assay, cells were harvested after 48 hours of transfection and stained with FITC and PI (BD Pharmingen). Cell assays were assessed by flow cytometry (Becton Dickinson), and FlowJo is used to analyze data.

### 2.5 Colony formation assay

1000 GC cells were plated on 6‐well plates, and the cells were washed three times with PBS and stained with crystal violet after 7 days. The cells were photographed and counted with ImageJ.

### 2.6 Cell migration assays

For the migration assay, 2 × 10^5^ cells were seeded in the top chamber with non‐FBS DMEM, and medium containing 10% FBS was added into the lower chamber. The cells were incubated for 24 hours. The non‐migration cells were wiped off with cotton swabs on the upper membrane surface. Then, the migrated cells on the lower membrane surface were stained with Hoechst 33 342 and counted under a microscope.

### 2.7 Cell invasion assays

Twenty‐four‐well, 8.0 µm transwell chamber (BD Biosciences) was coated with diluted Matrigel (BD Biosciences) in PBS and dried for 3 hours. For the invasion assay, 2 × 10^5^ cells were seeded in the top chamber with non‐FBS DMEM, and medium containing 10% FBS was added into the lower chamber. The cells were incubated for 24 hours. The non‐invasive cells were wiped off with cotton swabs on the upper membrane surface. Then, the invaded cells on the lower membrane surface were stained with Hoechst 33 342 and counted under a microscope.

### 2.8 Quantitative real‐time PCR

Total RNA was isolated from cells with TRIzol reagent (Invitrogen) following the manufacturer's instructions. For analysis of hsa_circ_101882 expression, cDNA was synthesized by qPCR RT Kit (Toyobo) as described by the manufacturer. SYBR Green kit (Qiagen) was used to perform qPCR. The β‐actin was used for internal control. The ABI Prism 7500 Sequence Detector (Applied Biosystems) was used to run real‐time PCR. The mRNA expression level was examined by the relative quantitative gene expression. The primers performed used are listed in Table 1.

**Table 1:** The primer sequences included in this study

Name	Primer sequences (5′‐3′)
hsa_circ_101882	
Forward	GTGTTCAAGAAATTGCAGAAATGCTTTC
Reverse	GCAAATGGTATTTCTGCTATCCAACAAGG
GAPDH	
Forward	GCACCGTCAAGGCTGAGAAC
Reverse	ATGGTGGTGAAGACGCCAGT

### 2.9 Western blot analysis

The MGC‐803 cells were seeded into 6‐well plates. Total protein was extracted from treated cells using IP lysis butter. Subsequently, the protein was subjected to SDS‐PAGE separation and electro‐transferation to PVDF membranes. Primary antibodies against E‐cadherin, N‐cadherin, vimentin, Snail, and GAPDH were purchased from Cell Signaling Technology. All the secondary antibodies were obtained from PerkinElmer, Inc.

### 2.10 Statistical analysis

All data are represented by the mean ± SD. Differences among the different treatment groups were assessed by Student's *t* test. The difference between data was considered to be statistically significant when *P* < .05 (*), to be very significant when *P* < .01 (**), and to be very much significant when *P* < .001 (***).

## 3 RESULTS

### 3.1 Hsa_circ_101882 is upregulated in both GC cell lines and clinical specimens

**Figure 1:**
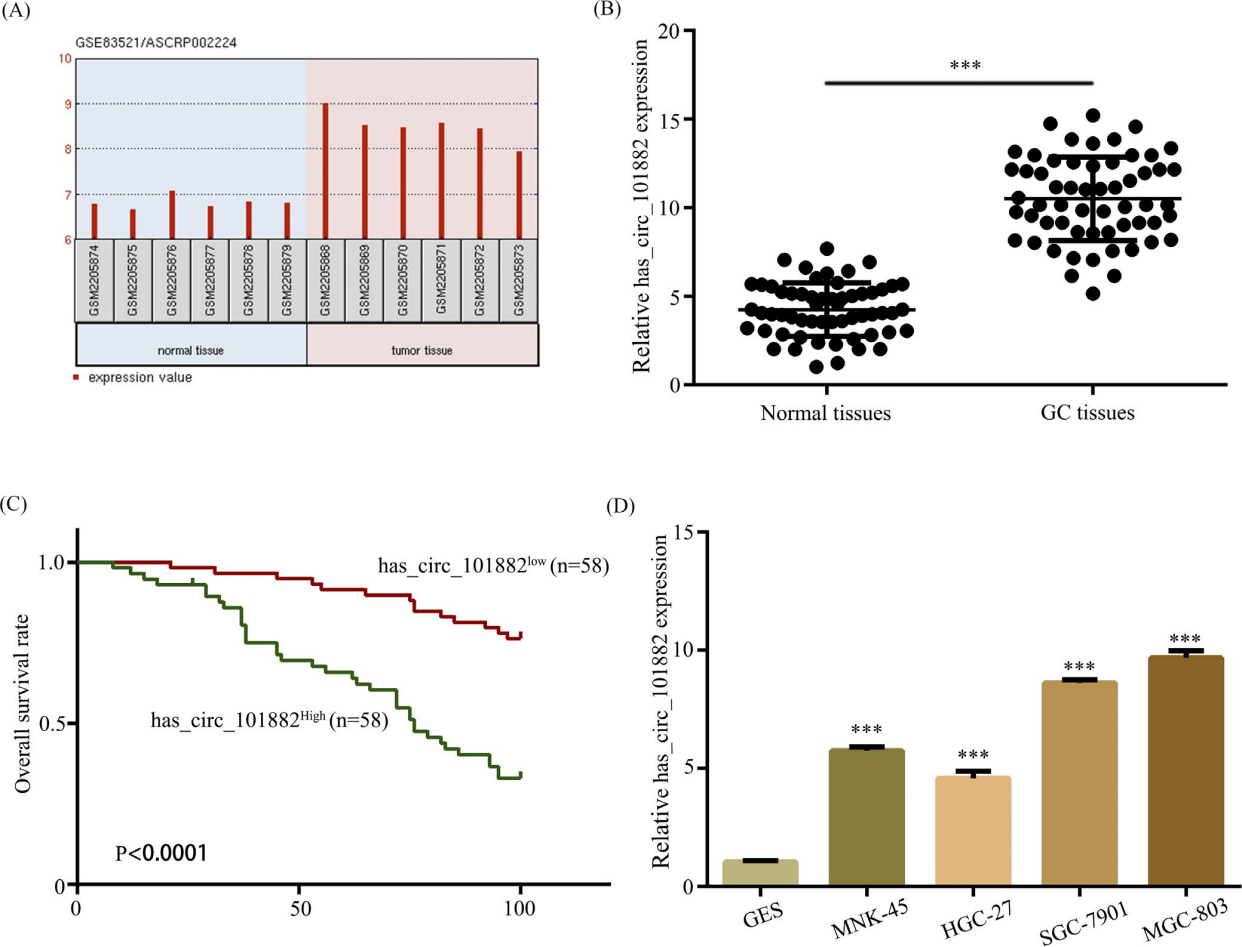
Hsa_circ_101882 is upregulated in both GC cell lines and clinical specimens. A, GEO dataset (GSE83521) showed that Hsa_circ_101882 was significantly increased in tumor tissues compared to non‐tumor tissues. B, The expression of hsa_circ_101882 was detected by RT‐PCR in human GC tissue and adjacent normal tissue. C, Kaplan‐Meier survival analysis. D, The expression of hsa_circ_101882 was detected by RT‐PCR in human GC cell lines MGC‐803, HGC‐27, SGC‐7901, and MNK‐45 and normal gastric mucosa cell line GES. The data shown represent the mean ± SD (n = 3). *P < .05, **P < .01, ***P < .001

We initially evaluated expression levels of circRNAs in GC by retrieving the microarray data in the GEO dataset (GSE83521), and normalized microarray data were analyzed by using GEO2R after applying log2 transformation. The microarray data showed hsa_circ_101882 expression was upregulated in tumor samples compared with normal samples. Thus, hsa_circ_101882 was chosen for further research (Figure 1A). In addition, the 58 GC tissues and corresponding adjacent normal tissues were obtained to examine hsa_circ_101882 expression. The results revealed that hsa_circ_101882 expression levels were significantly increased in GC tissues, as compared to corresponding adjacent normal tissues (Figure 1B). Kaplan‐Meier survival analysis revealed that higher expression of hsa_circ_101882 in patients with GC was associated with lower survival rate (Figure 1C). It yet showed the expression of hsa_circ_101882 was significantly increased in GC cell lines compared with GES cells (Figure 1D). Therefore, hsa_circ_101882 was upregulated in both GC cell lines and clinical specimens, which suggested its dysregulation may contribute to GC progression.

### 3.2 Downregulated of hsa_circ_101882 exerts a tumor suppressor function in GC

**Figure 2:**
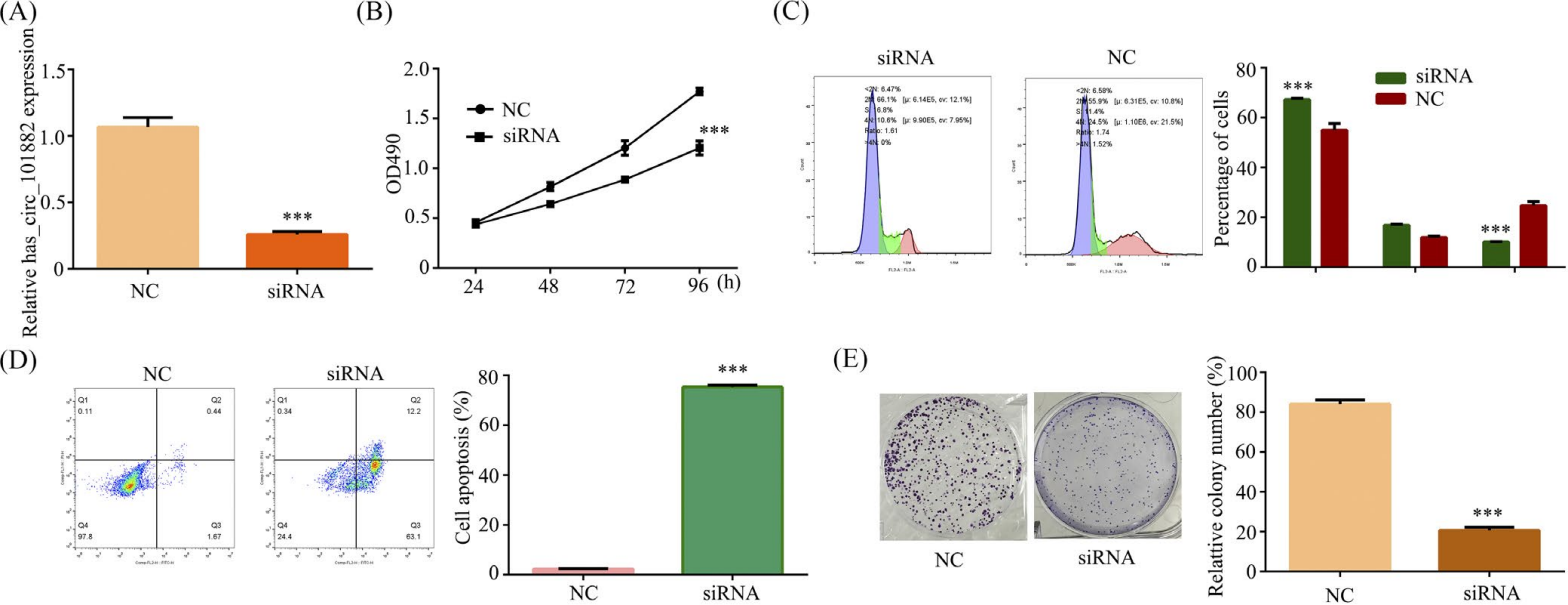
Downregulated of hsa_circ_101882 exerts a tumor‐promotive function in GC. A, The expression of hsa_circ_101882 was detected by RT‐PCR in MGC‐803 cell transfected with si‐hsa_circ_101882 or si‐NC. B, The tumor growth was detected by MTT assay in MGC‐803 cell transfected with si‐hsa_circ_101882 or si‐NC. C, The cell cycle progression was detected by flow cytometry assays in MGC‐803 cell transfected with si‐hsa_circ_101882 or si‐NC. D, The cell apoptosis was examined by flow cytometry assays in MGC‐803 cell transfected with si‐hsa_circ_101882 or si‐NC. E, The colony formation in MGC‐803 cell transfected with si‐hsa_circ_101882 or si‐NC was identified. The data shown represent the mean ± SD (n = 3). *P < .05, **P < .01, ***P < .001. All siRNA was si‐hsa_circ_101882

To verify that hsa_circ_101882 promotes GC cell growth, MGC‐803 cells were transfected the si‐hsa_circ_101882 or si‐NC control. The hsa_circ_101882 expression was decreased in transfected si‐hsa_circ_101882 MGC‐803 cell compared with si‐NC MGC‐803 cell (Figure 2A). In addition, transfected si‐hsa_circ_101882 or si‐NC MGC‐803 cell growth was examined by MTT assay. The result showed that cell growth was markedly inhibited in si‐hsa_circ_101882 MGC‐803 cells at 48 hours, 72 hours, and 96 hours, but no significant difference at 24 hours (Figure 2B), explained that hsa_circ_101882 promotes GC cell growth in vitro. Then, to examine the effect of hsa_circ_101882 on GC cell apoptosis and cycle progression, the flow cytometry was performed. The results showed that si‐hsa_circ_101882 transfection induced G1 arrest (Figure 2C) and promoted GC cell apoptosis (Figure 2D) compared with si‐NC transfection. Moreover, downregulated of hsa_circ_101882 greatly weakened colony formation ability of MGC‐803 cells (Figure 2E). Therefore, hsa_circ_101882 functioned was considered a tumor promoter in GC.

### 3.3 Downregulated of hsa_circ_101882 inhibited MGC‐803 cell migration and invasion in vitro

**Figure 3:**
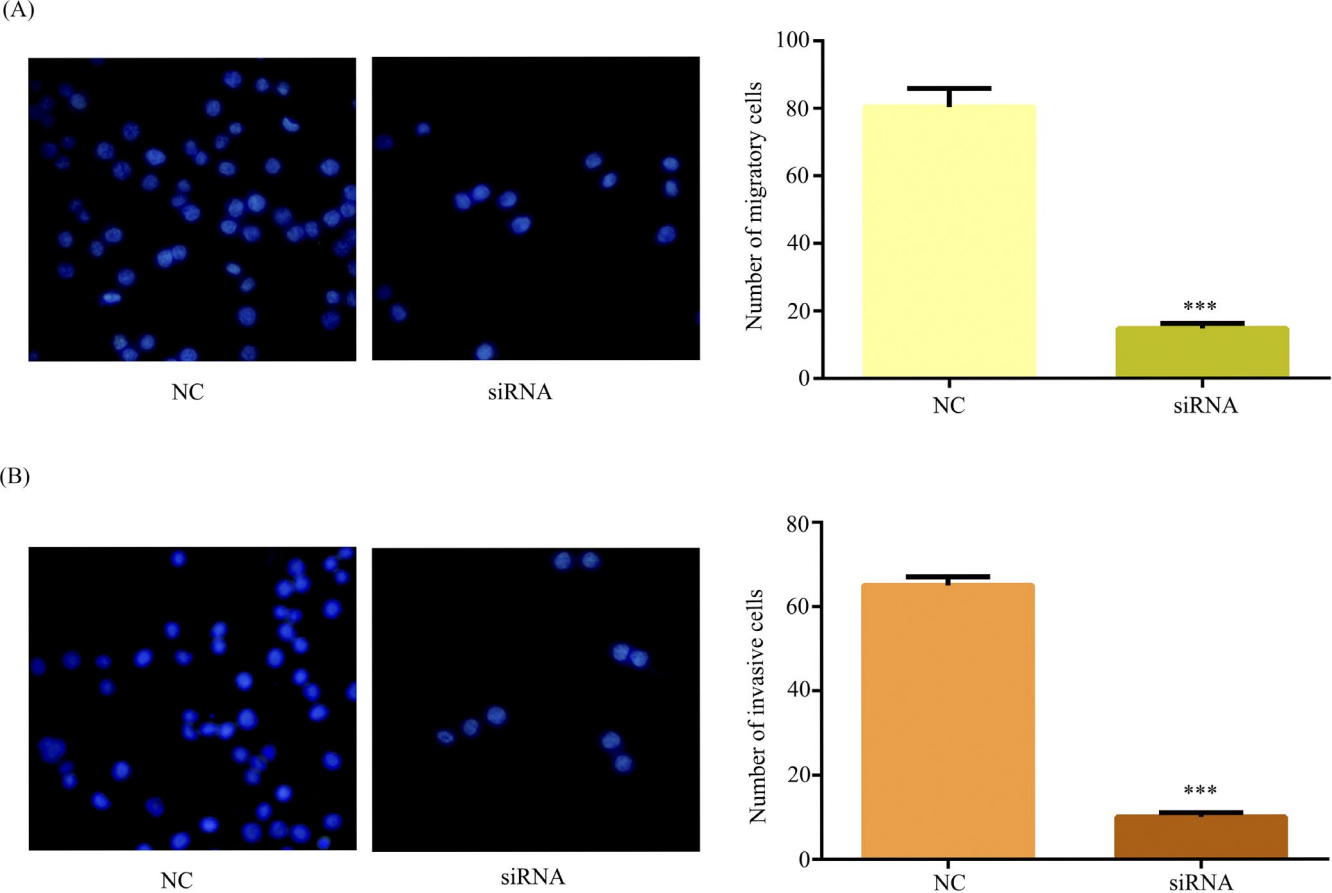
Downregulated of hsa_circ_101882 inhibited MGC‐803 cell migration and invasion in vitro. A, The transwell migration assay detected cell migration in MGC‐803 cell transfected with si‐hsa_circ_101882 or si‐NC. B, The transwell invasion assay detected cell invasion in MGC‐803 cell transfected with si‐hsa_circ_101882 or si‐NC. All data shown represent the mean ± SD (n = 3). *P < .05, **P < .01, ***P < .001. All siRNA was si‐hsa_circ_101882

Through clinical data, we found that high expression of hsa_circ_101882 was related to metastasis, explained that hsa_circ_101882 may promote GC metastasis (Table 2). To further verify hsa_circ_101882 promoted GC metastasis, the transwell assays were used to detect metastasis in MGC‐803 cells transfected si‐NC/si‐hsa_circ_101882. The result showed downregulation of hsa_circ_101882 expression could impede cell migration (Figure 3A) and invasion (Figure 3B) in MGC‐803 cells, suggesting that metastasis of GC was promoted by upregulating hsa_circ_101882 expression.

**Table 2:** Relationship between clinical features and hsa_circ_101882 expression in 30 GC patients

No. of variables	Cases	hsa_circ_101882 expression		*P*‐value
		Low (n %)	High (n %)	
Age (y)				.452
<55	17	9 (52.9%)	8 (47.1%)	
≧55	13	6 (46.2%)	7 (53.8%)	
Gender				.845
Male	15	8(53.3%)	7 (46.7%)	
Female	15	7 (46.7%)	8 (53.3%)	
TNM stage				<.01
I‐II	8	2	6	
III‐IV	22	4	18	
Tumor size				<.01
<5 cm	9	2	6	
≧5 cm	21	4	18	
Metastasis				<.01
No	10	8	2	
Yes	20	1	19	

### 3.4 Hsa_circ_101882 can modulate EMT of GC cells

**Figure 4:**
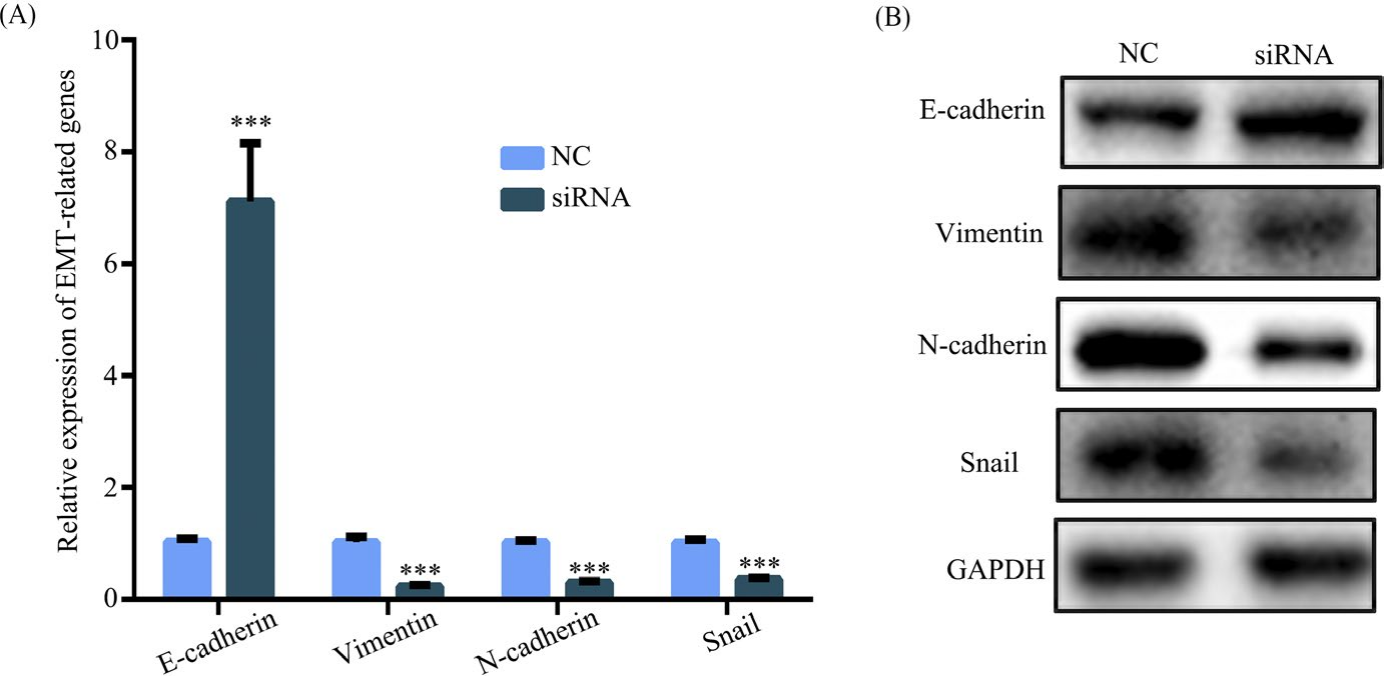
Hsa_circ_101882 can modulate EMT of GC cells. A, RT‐PCR was conducted to analyze the mRNA levels of E‐cadherin, vimentin, N‐cadherin, and Snail in MGC‐803 cell transfected with si‐hsa_circ_101882 or si‐NC. All data shown represent the mean ± SD (n = 3). *P < .05, **P < .01, ***P < .001. B, Western blot was conducted to analyze the protein levels of E‐cadherin, vimentin, N‐cadherin, and Snail in MGC‐803 cell transfected with si‐hsa_circ_101882 or si‐NC. All siRNA was si‐hsa_circ_101882

Above data have underlined that hsa_circ_101882 was associated with metastasis of GC. Then, the metastasis mechanism was further explored, and EMT had been considered to be associated with tumor metastasis. Thus, to examine effect of hsa_circ_101882 inhibition on EMT‐related genes, the RT‐PCR and Western blot were utilized. The result showed mesenchymal marker gene expression including vimentin, Snail, and N‐cadherin in mRNA and protein level were decreased in GC cell transfected with si‐hsa_circ_101882, while epithelial marker E‐cadherin was increased in mRNA (Figure 4A) and protein level (Figure 4B). Therefore, hsa_circ_101882 could activate EMT signaling pathway in GC progression.

## 4 DISCUSSION

Metastatic relapse, a complicated process including cell adhesion, migration, and reaching target organs, is one of the main causes of poor prognosis of GC. Although circRNA molecular mechanisms have been discovered to be associated with GC, research in this area is still rare.^8^, ^9^ Thus, studying the characterization of molecules mechanism in GC can provide more clues for understanding pathogenesis of GC. Notably, an excellent molecular marker circRNA plays an indispensable role in GC.^10^, ^11^, ^12^, ^13^, ^14^, ^15^, ^16^, ^17^

Recently, it has been reported that circRNA is associated with tumor development, migration, and invasion, for example, circRNA_001569 targeting miR‐145 in the proliferation and invasion of colorectal cancer.^6^ Circular RNA CCDC66 promotes colon cancer growth and metastasis.^7^ Overexpression of circRNA_100876 in non–small‐cell lung cancer and its prognostic value.^18^ Notably, circRNA has been found to play an important role in GC development, migration, and invasion. For instance, circRNA_100269 is downregulated in gastric cancer and suppresses tumor cell growth by targeting miR‐630.^8^ CircRNA_001569 promotes cell proliferation through absorbing miR‐145 in gastric cancer.^6^ Hsa_circ_0000673 is downregulated in gastric cancer and inhibits the proliferation and invasion of tumor cells by targeting miR‐532‐5p.^19^ Downregulation of circPVRL3 promotes the proliferation and migration of gastric cancer cells.^20^ In addition, circRNAs contribute to gastric cancer tumorigenesis through sponging miRNA. For example, circular RNA circCACTIN promotes gastric cancer progression by sponging miR‐331‐3p and regulating TGFBR1 expression.^21^ A novel circular RNA hsa_circ_0008035 contributes to gastric cancer tumorigenesis through targeting the miR‐375/YBX1 axis.^22^ Circular RNA AKT3 upregulates PIK3R1 to enhance cisplatin resistance in gastric cancer via miR‐198 suppression.^23^ However, it is basically no research report on hsa_circ_101882 in GC. Therefore, our research revealed the role of hsa_circ_101882 in GC and provides anti‐GC research for future treatment of GC.

In this present study, hsa_circ_101882 was the first report in GC patients. We found that hsa_circ_101882 expression levels were significantly increased in GC cell lines and GC tissues compared with control. Besides, function assays revealed that hsa_circ_101882 could promote GC cell proliferation, migration, and invasion and inhibit cell apoptosis. Our result strongly suggested that hsa_circ_101882 plays a promoting role in GC progression and metastatic.

Epithelial‐to‐mesenchymal transition is a critical cellular progress in cancer metastasis.^24^ Recent reports have indicated circRNAs play a significant role in the EMT regulation.^25^ In GC, circRNA_0023642 promotes migration and invasion of gastric cancer cells by regulating EMT.^26^ In this present study, by RT‐PCR and Western blot, we found hsa_circ_101882 knockdown decreased mesenchymal marker gene expression including vimentin, Snail, and N‐cadherin in mRNA and protein level in GC cell, while epithelial marker E‐cadherin was increased in mRNA and protein level, implying that hsa_circ_101882 enhanced activation of EMT signaling pathway in GC.

Based on the above results, it is strongly suggested that hsa_circ_101882 markedly promotes migration and invasion by regulating EMT in GC. Therefore, it is necessary and valuable to further research the mechanisms of how hsa_circ_101882 regulated EMT and make hsa_circ_101882 transform a clinical marker.

## CONFLICT OF INTEREST

The authors declare no conflict of interest.
